# Cataract Operations—A Clinical Lecture

**Published:** 1886-03

**Authors:** A. W. Calhoun

**Affiliations:** Professor of Diseases of the Eye, Ear and Throat, Atlanta, Ga.


### CATARACT OPERATIONS.

A CLINICAL LECTURE DELIVERED AT THE ATLANTA MEDICAL COLLEGE BY A. W. CALHOUN, M. D., PROFESSOR OF DISEASES OF THE EYE, EAR AND THROAT, ATLANTA, GA.

Reported for THE ATLANTA MEDICAL AND SURGICAL JOURNAL.

GENTLEMEN—I have the opportunity of showing you to-day two modes of operating for cataract: one of the cases I show you is a man sixty-five years old, and is denominated hard cataract, while the other is in a girl six years old and is a soft cataract. The hard cataract occurs in elderly persons, or, more properly speaking, in persons over thirty-five years old. The soft cataract occurs in young persons from infancy to thirty or thirty-five years old.

The lens naturally hardens as persons grow older, and the disease constituting cataract hardens it still more, so that by the time the cataract is matured the nucleus of the lens becomes quite hard and opaque and the cortical substance more or less hard.

By soft cataract we mean that opaque condition of the lens where the cortical substance, and the nucleus as well, remain soft, even after it is fully matured or ripened.

There are two methods of operating, that for the hard cataract being called extraction, and for the soft, discision or laceration. By extraction is meant the entire removal of the lens at one operation, and by discision or laceration, that the lens is allowed to remain and is absorbed by being broken up.

The method of operating I usually employ, and the one I shall make on the patient before you with hard cataract is known as Grafe's extraction.

There is a certain preparatory treatment for these operations that should not be neglected. A patient should always be given a cathartic the night before the operation, so that the bowels may be well emptied, as they will of necessity be kept perfectly quiet for a number of days after the operation. Hence I always advise that my patient take a full dose of sulphate of magnesia the night before operating. On the morning of the day for the operation, a two-grain solution of sulphate of atropia should be dropped into the eye until the pupil is fully dilated, as is the case with the patient before you. When everything is ready the patient should be placed in the bed he will occupy while under treatment, and the operation made while he is in bed. This is done to avoid any possible injury to the wound in the eye by carrying the patient from one room to another.

In the case before you we cannot do this; we must operate here in the amphitheater in order that you may witness it, but I advise you against this as a practice. In private work I always operate on the patient in bed, and do not allow them to be moved for several days.

Opinions differ very widely as to the question of anæsthetics in the operation. I prefer never to give a patient chloroform or ether in operating for extraction. The danger of severe vomiting following the use of them, and the loss of more or less of the vitreous humor through the opening made for the exit of the lens is too great. My opinion as to the use of cocaine in cataract extractions has undergone recently a complete change. It relieves the pain of the operation absolutely, and quiets the fears of the most timid patients, but the results in many instances are anything but satisfactory. The use of even a weak solution of cocaine causes decided contraction of the conjunctival and sub-conjunctival blood-vessels and this diminishes the amount of nutrition to the cornea. When the wound is made for the removal of the lens, the cocaine comes in contact with the cut surfaces of the cornea and also passes into the anterior chamber, and in the course of twelve to twenty-four hours violent inflammation, with more or less sloughing of the cornea, ensues. Even where this does not occur, iritis is very apt to follow with very profuse effusion of lymph in the pupiliary space and posterior chamber. For these reasons I advise you against the use of cocaine in cataract extractions.

The operation is not a very painful one, and there are very few patients who will not gladly undergo the operation with the expectation of having their sight restored.

In the case before us we will use no anæsthetic. The patient is placed on the table and the lids separated with an eye speculum; I now grasp the conjunctiva with fixation forceps just below the lower corneo-sclerotic margin. This is done in order to securely steady the eye. I enter a Grafe's knife at the upper corneo-sclerotic margin into the anterior chamber and push the point across the anterior chamber in front of the iris and bring it out at a point corresponding to that at which it entered; by a see-saw motion of the hand I cut the flap upwards, making the cut as near in the corneo-sclerotic junction as possible. After this I make an iridectomy; in doing this I cut a sufficiently large portion out to allow an easy delivery of the lens. Now with the cystotome the anterior capsule is ruptured and with light but steady pressure on the lower portion of the ball, the upper edge of the lens will tilt forward and slide out of the ball, as you see it now doing.

The dressing consists in cleansing the wound thoroughly of all cortical substance and of freeing it of any portion of iris which may have remained in it; the eye is then bandaged and the patient sent to his room. My rule is to dress the eye morning and night, using a few drops of atropia solution each time to keep the pupil well dilated.

As a general rule, if the patient progresses satisfactorily for three or four days, there is then very little danger to be apprehended from the operation; if, on the other hand, he should begin to complain of much pain and the lids should become swollen, then I know that there is some complication to contend with; either the iris or cornea is involved; when such is the case they must be combatted with appropriate means. At the end of the fifth or sixth day I will allow this patient to sit up if no complication has appeared by that time, and at the end of the tenth day the bandage will be substituted with smoked glasses and the patient allowed to expose himself to the light gradually.

In making the operation for soft cataract, there is no reason why an anæsthetic should not be used if desirable. There is no objection to the use of cocaine in this operation as in the operation for extraction.

In the case before you we will use cocaine; the speculum and forceps are used as in the operation by extraction; with a cataract-needle I penetrate the cornea far to one side of the pupil and bring the point in contact with the anterior capsule at its upper border and make a slit downwards; now another slit is made at right angles to the first one; this allows the aqueous humor to come in contact with the lens substance and it is slowly absorbed. The pupil must be kept dilated and in three or four weeks the operation will be repeated. This is repeatedly done until the lens is entirely absorbed. Usually two operations is all that is required to bring about the desired result.

After either operation it will be necessary to adjust cataract glasses to the eyes; this should be done in the case of hard cataract in three or four weeks; in the other it will require eight or ten weeks.

---

Tolerance of Morphine in an Infant.—A correspondent, from Winnipeg, writes: "I have a case of spina bifida, with hydrocephalus, in a child four and a half months old. The spinal deformity includes the last three lumbar vertebræ. There is complete paralysis of all parts below the seat of lesion, including the sphincters, and also double talipes varus. The child, a little girl, has seemed to suffer excruciating pain ever since birth, keeping up a continual screaming. As we hoped death would soon terminate her troubles, no operation or treatment of any kind has been instituted, except the use of narcotics to allay pain. At the present date she takes half a grain of sulphate of morphia a day, and this has only a quieting effect. She takes one-sixth of a grain for a dose, and is actually thriving on it, so that I am afraid some operation will have to be performed, if only to give her a chance of dying."—*Medical Record.*

